# Flash vacuum expansion, a low-cost and energy-efficient alternative process to produce high-quality fruit puree: Application to *Physalis peruviana*

**DOI:** 10.1016/j.heliyon.2023.e16969

**Published:** 2023-06-18

**Authors:** Claudia Arias, Pablo Rodríguez, Iris Soto, Rowan Vaillant, Misael Cortés, Fabrice Vaillant

**Affiliations:** a Universidad Nacional de Colombia, Medellín, Facultad Ciencias Agrarias (Departamento Ingeniería Agrícola y Alimentos), Medellín (Antioquia), Colombia; b Corporación Colombiana de Investigación Agropecuaria-Agrosavia, Centro de Investigación La Selva, km. 7, vía Rionegro - Las Palmas, Sector Llanogrande, Rionegro-Antioquia, (Research Unit ITAV: Innovaciones Tecnológicas para agregar Valor a Recursos Agrícolas), Agrosavia, Colombia; c Universidad de Costa Rica (UCR), Chemical and Environmental Engineering, Ciudad Universitaria Rodrigo Facio, Código, Postal 11501-2060, San José, Costa Rica; d French Agricultural Research Centre for International Development (CIRAD), UMR Qualisud, Rionegro (Antioquia), Colombia; e Joint Research Unit—UMR Qualisud, Univ Montpellier, Avignon Université, CIRAD, Institut Agro, IRD, Université de La Réunion, Montpellier, France

**Keywords:** *Physalis peruviana*, Functional foods, Flash vacuum expansion, Innovative technology, Shelf life

## Abstract

Goldenberry has great potential for the development of high-quality products due to its attractive sensory attributes, bioactive compounds, and health benefits. However, postharvest losses are high due to the lack of processing technologies that can both be adapted to rural conditions in producing countries to generate high-quality products. Flash vacuum expansion coupled with vacuum pulping is a new process that can meet these requirements. In the process, the steam holding time (30, 40, and 50 s/130 kPa) and flash vacuum expansion (5 ± 1.2 kPa) were studied. The logarithmic reduction of microbial load and some quality indicators were analyzed during the process and during storage to assess the shelf life of fruit purées. The FVE process with 40 s steam blanching led to a microbial reduction of over 6 log colony forming units (CFU)/g, increased yield and β-carotene content, and preserved most of the AA content (4–12%). Based on the half-lives of the quality indicators, the shelf life of the purées was between 16 d (20 °C) and 90 d (4 °C). The energy consumption was estimated at approximately 0.30 kWh/kg of product. These results demonstrate that the FVE process, although it includes heat treatment, allows a short exposure to heat of the whole fruits to obtain a high-quality puree with an adequate shelf life in a single step, with a relatively low equipment investment and moderate energy consumption.

## 1 Introduction

Goldenberry (*Physalis peruviana*) is an exotic fruit belonging to the Solanaceae family native to South America. *Physalis peruviana* has been given multiple names around the world; in Colombia, it is known as “uchuva”, and in English-speaking countries, it is known as “goldenberry” or Cape gooseberry. The fruit is a berry contained in a shell called a calyx. The fruit has a diameter between 1.25 and 2.50 cm, an average weight of 4–10 g, a smooth, waxy, orange-yellow skin, and juicy pulp containing numerous small seeds [1]. Its production extends throughout the South American Andes. There is great interest in the consumption and industrial use of this fruit for its nutritional and medicinal properties [2,3]. Recently, its functional value for health has been published, and insulin signaling has been shown to reduce the risk of type 2 diabetes [4].

Colombia is one of the main exporting countries in the world (approximately 7800 tons/1700 ha in 2021), mainly to the European and US markets. The shelf life of fresh fruits is strongly affected by fungal growth, loss of firmness, and weight loss during storage [5,6]. After harvest, between 10 and 15% of fruits are discarded due to cracking or other visual defects. Within the export chain, postharvest losses are estimated to be between 28 and 43% [7]. Therefore, the processing of fruits rejected from the export market is fundamental to increasing the competitiveness of the value chain, which requires the development of viable innovative technologies at the level of small and medium agroenterprises (SMAEs). In developing countries, production is dispersed in different areas at long distances to large enterprise facilities. SMAEs need innovative processes to obtain high-quality products with an appropriate shelf life to reach diversified markets.

Among the innovative technologies that have been studied for the transformation of goldenberry is high hydrostatic pressure (HHP) [3,8,9]. However, the implementation of HPP in developing countries is difficult due to the very high investment costs of SMAEs.

The principle of the Flash vacuum expansion (FVE) process is based on the disintegration of plant tissues by the combined effect of blanching and the application of a sudden vacuum. The raw material is placed in a heating chamber with saturated steam at 130 kPa and then quickly subjected to partial vacuum pressure (2–5 kPa) in an expansion chamber. This expansion generates the instantaneous evaporation of a part of the water contained in the plant tissues, causing the cells to burst [10]. The FVE process coupled with pulping and packaging could allow the development of high-quality goldenberry purées with an adequate shelf life. The process involves short heating times, and the instantaneous application of vacuum could rapidly cool the product. In this way, high energy efficiency is achieved at a low cost. Therefore, the aim of this work was to evaluate the effect of different blanching holding times and vacuum applications on heat transfer, quality, shelf life, and energy consumption during goldenberry purée production.

**Table:** Description of the abbreviations in the article.

Nomenclature	Subtract
D: cross-sectional diameter of fruit (m)	p Fruit purée
Cp: specific heat of the fluid (J.kg^−1^. K^−1^)	f fruit
λ: Thermal conductivity (W.m^−1^.K^−1^)	steam
P: pressure (Pa)	k number of subsystem
q: effective heat flux (kW.m^−2^)	t time
Thermal diffusivity = m^2^.s^−1^	tp time of operation
ρ: Specific mass (kg.m^−3^)	w liquid water
ΔH_fg:_ change of enthalpy	0 Ambient temperature
T: time	sb Steam blanching
ᶯ: Efficiency of the boiler (80%)	st storage
λ:Thermal conductivity (W.m^−1^.K^−1^)	
Q: heat source (W.s^−1^)	
m: mass flow (kg.h^−1^)	
T: temperature of fruit or purée (°C)	
H_fg_: The specific enthalpy of evaporation of steam (KJ/kg)	
κ: rate constant of 1st order kinetic reaction (day^−1^)	
t ½: half-life time	
ΒΙ: Browning index	
${\dot{m}}_{steam}$: Steam flow (kg.h^−1^)	
*μ*: Velocity of surface m.s^−1^	

## 2 Materials and methods

### 2.1 Vegetal material

The fruits used were goldenberry discarded from the export chain according to the export classification criteria of the Caribbean Exotic packing house (Rionegro, Antioquia-Colombia). These fruits were harvested in August 2021 from orchards certified in Global good agricultural practice (GAP) (Rionegro, Antioquia-Colombia). Fruits were sorted at a maturity stage between 4 and 6, which correspond to light orange, orange, and intense orange skin colors, respectively NTC 4580 [11]. Before the process, the calyx was manually removed, the fruits were washed, and unripe fruits (green color of the skin) and foreign materials were eliminated. Goldenberries were stored at refrigeration temperature (4 °C) until FVE processing.

### 2.2 Flash vacuum expansion (FVE) process

Goldenberry purées were obtained using a pilot line of FVE constituted by a cylindrical stainless-steel chamber (Ø = 154 mm; h = 175 mm, v = 6 L), connected to an expansion chamber (volume = 37.5 L), coupled to a rotating pulper/finishing. Between the heating and vacuum chambers, there is a large opening diameter ball valve (ø = 150 mm), which allows the passage of the material using a fast pneumatic actuator (80% opening of the valve in 1 s). The vacuum was generated by a liquid ring pump (Robuschi RVS_3 M − 02, Parma, Italy) capable of delivering a gas extraction rate of 4200 m^3^/h, providing a vacuum pressure of 5 ± 1.2 kPa inside the expansion chamber. Water vapor is condensed through a heat exchanger to limit the volume of gas suctioned by the vacuum pump. The vacuum pressure was recorded by a digital vacuum transducer (Sitrans P, Siemens, Germany). Inside the vacuum chamber, a rotary pulping system (1500 rpm/30 s) was set up to sieve the fruit puree on 1-mm meshes. Two aseptic tanks allow the recovery of sifted fruit purées and coproducts such as seeds and large fragments. The whole pilot line of FVE was steam-sterilized before the process and between each treatment. The vacuum was broken through a vented sterilizing filter with an absolute air particle removal rate of 0.003 μm (Emflon® PFR Filters, Pall, Washington, NY, USA).

Whole goldenberry fruits (1.5 kg per batch) were introduced into the steam heating chamber, and then an application of vacuum at 5 kPa/5 s was performed for initial degassing. Then, steam previously filtered with a culinary filter (0.2 μm pore diameter, PALL, Washington, NY, USA) was injected directly, maintaining a hydrostatic pressure of 130 kPa inside the heating vessel for different holding times of 30, 40, and 50 s. During blanching, a valve at the bottom of the heating tank remained partially open to permanently evacuate the condensates and promote a minimum flow of steam. After achieving the holding time target, the steam and condensate valves were closed, and the pneumatic ball valve was suddenly opened. The fruits then passed immediately by gravity into a vacuum expansion chamber undergoing an instantaneous pressure drop to 5 kPa.

**Fig. 1:**
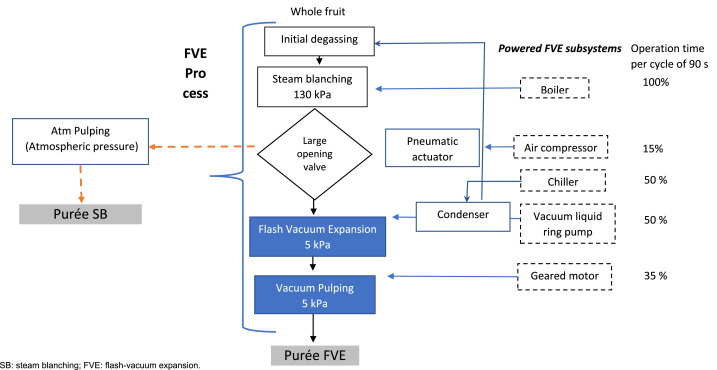
Flow diagram of the flash vacuum expansion process and powered subsystems.

When only steam blanching (SB) was performed, the fruits fell into the same chamber, and the mash was sieved, but this all occurred without implementing vacuum suction (Fig. 1). Finally, the sieved purée that fell into the storage tank could be pressurized to atmospheric pressure independently of the previous circuit through the vented air-sterilizing filter for the next ultraclean packaging step. The obtained purée was immediately packaged in previously irradiated multilayer bags (plasticized, PET/Foil/LDPE 120 μm, Smurfit Kappa®, Dublin, Ireland) using a bag-in-box semimanual filler (Sympaty ROp 320, Technibag, Villefranche-sur-Seine, France) set up inside a laminar flow hood. Four replicates were made for each treatment. Notably, the experiments were carried out at 2100 m.a.s.l. in AGROSAVIA's pilot plant in Rionegro (Antioquia, Colombia), corresponding to an average atmospheric pressure of approximately 80 kPa.

### 2.3 Study of heat transfer

In the heating chamber, the temperatures of the environment and the fruits were recorded using probes (DMCA-1019-2, Maycin, Medellín, Colombia). The temperature inside the central region of 3 fruits, placed in the middle of the whole fruit mass, was detected by flexible probes (diameter 1 mm). Heat transfer was simulated by COMSOL (Multiphysics v5.6) considering a goldenberry fruit as a sphere [12] with an average equatorial diameter of D = 17.5 × 10^−3^ m. The geometry was simulated as a 2D axis-symmetric sphere. In the absence of reliable published thermal properties of *Physalis peruviana*, data from tomato, another Solanacea, were chosen to approximate heat transfers with heat capacity Cp = 2.750 kJ kg^−1^. K^−1^ and λ = 0.5 W m^−1^. K^−1^ (Ekpunobi, U. et al., 2014). The specific mass (ρ = 1100 kg·m^−3^) was reported for *Physalis peruviana* by Oliveira et al. (2016) [13].

The heat transfer equation (1) was used for the COMSOL simulation.

1 $$\rho \;C_{p}\frac{\delta T}{\delta t}+\rho C_{p}u\nabla T=\nabla .\left(\lambda \nabla T\right)+Q$$

For the simulation, the steam was assumed to be renewed at such a high rate that the surface of the fruit in the chamber is instantly at the same temperature as the incoming steam. Moreover, since the fruit of interest is in the center of the chamber, direct contact with the vapor is negligible, which allows heat transfer to be applied by conduction alone without the need to add convection.

### 2.4 Physicochemical analysis

The yield was determined by the ratio of the mass of fruit purée obtained after sieving relative to the total mass of treated fruit. The pH, titratable acidity, and soluble solids were analyzed according to the standard methods of the AOAC (2005) (No 981.12, 942.15, and 932.12, respectively) [14]. The color was determined using a Color Flex EZ spectrophotometer (Hunter Lab, illuminant D65, observation angle 10°, Reston, VA, USA), and the results were expressed in the CIELAB color space. L* represents brightness or dullness in the range of 0–100, and a* and b* represent redness (+)/greenness (−) and yellowness (+)/blueness (−), respectively. The values of the total color differences (ΔE*), chroma (Cab), and hue (h°ab) were determined using equations 2, 3, 4):

2 $$\Delta E*=\sqrt{{L*-L_{0})}^{2}+{\left(a^{*}-{a*}_{o}\right)}^{2}+{\left(b*-b_{0}\right)}^{2}}$$

3 $$C_{ab}={\left(a^{*2}+b^{*2}\right)}^{\frac{1}{2}}$$

4 $$h_{ab}=\tan^{-1}\left(\frac{b^{*}}{a^{*}}\right)$$

Where L*_0_, a*_0_, and b*_0_ represent the initial values of L*, a*, and b*, respectively, immediately after processing at 0 days of storage. The browning index (BI) is calculated from the CIE L*a*b* coordinates by applying the equation given by Buera et al., 1986 [15], using equations 5, 6):

5 $$BI=\frac{100\;\left(x-0.31\right)}{0.17}$$

where x:

6 $$x=\frac{{(a}^{*}+{1.75L}^{*})}{({5.645L}^{*}+a^{*}-{0.012b}^{*}}$$

### 2.5 β-Carotene

Extraction was performed following the method published by Etzbach et al. (2018) with some modifications [16]. Briefly, approximately 1 g of sample was mixed with 10 mL of extraction solution (hexane: acetone 60:40); the mixture was centrifuged (3500 rpm, 5 min, 4 °C), and the supernatant was collected. Finally, the liquid obtained was filtered (0. 45-μm polyvinylidene fluoride (PVDF) membrane, syringe filter). The quantification was performed in a high-performance liquid chromatograph (HPLC) (Prominence UFLC 20A, Shimadzu, Kyoto, Japan) equipped with a photodiode array detector (PDA) and a Luna® C18 Column (2) 100 Å (250 mm* 4,6 mm ID* 5,0 μm) (Phenomenex Luna, Torrance, CA, USA).

Carotenoids were eluted using a mobile phase consisting of acetonitrile:methanol:acetone (60:30:10) at a flow rate of 1.2 mL/min and separated on a C18 column. The chromatograms were processed at 450 nm, and carotenoids were quantified as β-carotene equivalents using an external calibration curve (β-carotene standard (Sigma Aldrich C4582, Burlington, MA, USA) (0.4–0.01 mg/mL) (retention time 19.52 min). The calibration curve and validation parameters (inter and intra-day reproducibility) are shown in the supplementary content (see Figure S1 and Table S1). The results are expressed as mg of β-carotene/100 g of fresh weight (FW).

### 2.6 Ascorbic acid

The extraction and identification of ascorbic acid were carried out according to the methodology described by Lee et al. (2016) with some modifications, as described below [17]. Approximately 3 g of purée was weighed, 20 mL of a KH_2_PO_4_ solution (0.02 M, pH: 3.06 adjusted with 85% *ortho*-phosphoric acid) was added, and the mixture was stirred at room temperature (20 °C) for 1 min at a speed of 3000 rpm (Analog vortex mixer, VWR; Avantor delivered by VWR, Atlanta, GA, USA). Subsequently, the purée mixture was centrifuged at 4 °C for 15 min at 3000 rpm, and the supernatant was filtered using a 0.45-μm PVDF syringe filter. Identification and quantification were performed by HPLC (Prominence UFLC 20A, Shimadzu, Kyoto, Japan) coupled to a Prominence SPD-M20A diode array detector with a Luna® C18 Column (2) 100 Å (250 mm* 4.6 mm ID* 5.0 μm) and with a mobile phase of the same extraction solution.

The analysis conditions were mobile phase flow rate 1.0 mL/min, temperature 35 °C, injection volume 20 μL, absorption wavelength at 244 nm, and isocratic mode. The concentration of ascorbic acid in the purées was determined by the external standard method using an ascorbic acid standard curve (Sigma Aldrich 47863) (0.1–50 μg/mL), and the retention time was 4.37 min. The calibration curve and validation parameters (inter and intra-day reproducibility) are shown in the supplementary content (see Figure S2 and Table S2). The results were expressed as mg ascorbic acid/100 g purée on fresh weight (FW).

The degradation rates of ascorbic acid (AA) and β-carotene were fitted to first-order reaction kinetics, and the equation for this model is using equation (7).

7 $$A_{t}=A_{0}*\exp\left(-\kappa t_{st}\right)$$

where A_0_ is the initial content, and A_t_ is the content after storage time. The half-life (t_1/2_) is the time needed for 50% degradation of AA or β-carotene and was calculated using equation (8).

8 $$t_{1/2}=\mathrm{Ln}\;2/k$$

### 2.7 Microbiological analyses

Culture media were used by the deep sowing method on Petri dishes. Purée (10 g) was mixed with 90 mL of sterile peptone water (0.1% w/v). A tenfold dilution series was prepared in sterile peptone water for plating. The following culture media and conditions were used to enumerate the microbial cells: 1. Mesophilic aerobic bacteria count (DEV nutrient agar, Merck, Rahway, NJ), incubated at 37 °C for 2 days; 2. Mold and yeast (Sabouraud 4% dextrose agar, Merck), incubated at 25 °C for 5 days; 3. Fecal and total coliforms (Chromocult medium agar, Merck) were incubated at 37 °C for 2 days. All analyses were performed in triplicate. The results are reported as log CFU/g of the sample FW.

All the above was done for the experimental design previously described in Section 2.2 (Experiment 1). To determine the maximum logarithmic reduction of FVE, a batch of fruit was left for several days at room temperature until it reached a high level of contamination and processed at a heating time of 30 s with and without flash explosion (Experiment 2).

### 2.8 Analysis of energy consumption

The energy performance was estimated by analyzing the consumption of all the subsystems. The process consumes electrical energy to power the liquid ring vacuum pump, the air compressor that moves the pneumatic valve actuator, the pulper and the chiller for the production of cold water. Additionally, there is a consumption of thermal energy related to the generation of the steam required during the blanching of whole fruit. Thus, the total energy consumed during the FVE process is the sum of electrical power and thermal energy (equation (9)):

9 $$E_{Tot}=E_{electricity}+E_{steam}$$

The electrical energy consumed $E_{k}$ of each of the *k* subsystems of the abovementioned equipment is given by equation (10).

10 $$E_{k}=\int_{0}^{t_{0,k}}{\dot{W}}_{k}dt$$

The thermal energy required to produce steam $E_{steam}$ used in the direct steam injection system is given using equations 11, 12, 13, 14):

11 $$E_{steam}=\frac{{\dot{m}}_{steam}\left(\int_{T_{0w}}^{T_{v}}C_{p}\left(T\right)dT+\Delta H_{v}\right)}{\eta }$$

The thermal energy required for heating the fruit is given by:

12 $$Q=\frac{m_{f\;C_{p_{f}\Delta T}}}{t}$$

The mass flow of steam required to heat the fruit is given by:

13 $${\dot{m}}_{steam}=\frac{Q}{H_{v}}$$

Finally, the specific energy consumption $S_{E}$ is given by:

14 $$S_{E}=\frac{E_{Tot}}{m_{p}}$$

### 2.9 Statistical analysis

Holding times in the heating chamber and vacuum expansion (FVE) were used as factors in the full factorial (3x2) randomized experimental design. Heating-up times of 30, 40, and 50 s were applied with or without flash vacuum expansion. The treatments were defined as steam blanching (SB) for heating without vacuum application and flash vacuum expansion (FVE) when vacuum was applied after each heating-up time. Four replicates were tested to guarantee an experimental design power of 92% (Design-Expert 11, Stat-ease Inc.).

All quality analyses were performed in triplicate and randomized, and the data are expressed as the mean ± standard deviation (SD). Before analysis of variance (ANOVA), normality and homoscedasticity were analyzed (Shapiro-Wilk normality and Levene homoscedasticity tests, respectively). Then, ANOVA and Tukey's (*p* < 0.05) test were performed to assess significant differences between treatments. For the shelf life, a repeated-measures ANOVA was established (*p* < 0.05). Nonlinear regression analysis was performed according to the first-order model equation, and the goodness of fit was estimated (R^2^). Data analysis was carried out using the statistical software XLSTAT 2022.1.2 (Addinsoft).

## 3 Results and discussion

**Fig. 2:**
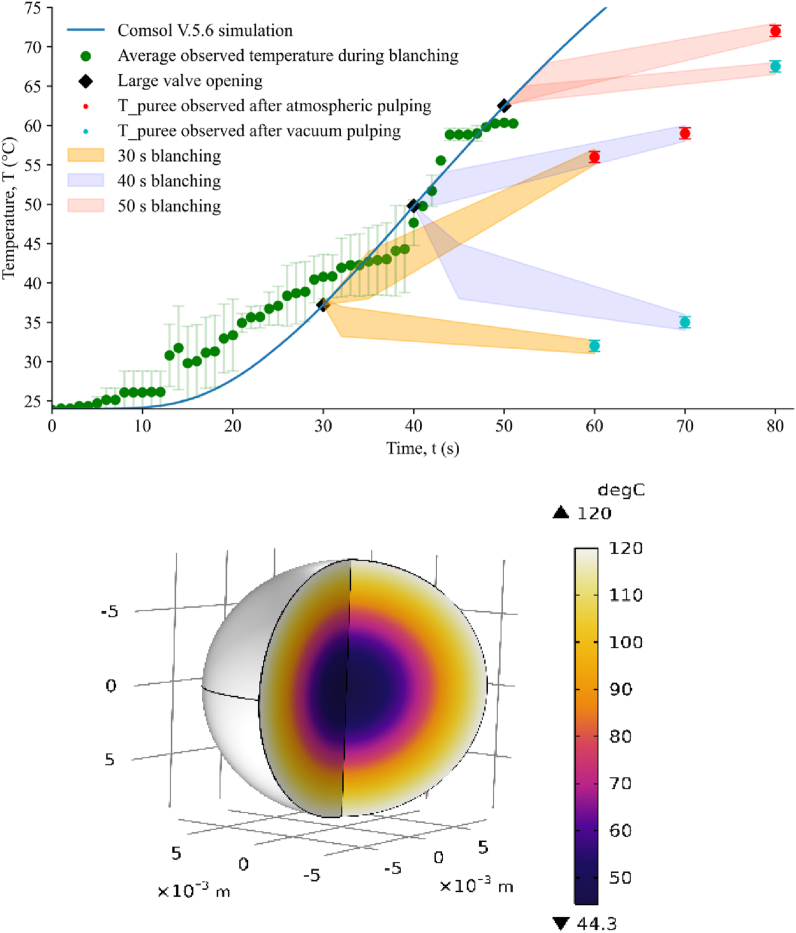
**a.** Average temperature profile at the center of fruits during different steam blanching (SB) holding times with or without flash vacuum expansion (FVE), and **b.** Simulation of the average temperature of the cross section of an average fruit after 40 s of steam blanching at 130 kPa.

Fig. 2a shows the average temperature profile recorded by thin probes in the central region of fruits (green dots) and the temperature of purées obtained after atmospheric or vacuum pulping (red and blue dots, respectively). The application of instant vacuum after steam blanching led to quick cooling. When pulping was performed at atmospheric pressure, an increase in the temperature of the purées obtained was observed because of the homogenization effect (Fig. 2a, red dots), and the final purée remained relatively hot for several minutes before cooling. At 50 s of holding time during steam blanching, no cooling was observed after FVE. Under these conditions, the fruits appeared totally destructured and stunted without turgidity because much liquid had flowed out, probably limiting the cooling effect of flash expansion.

A simulation of the blanching operation by COMSOL Multiphysics shows a relatively good agreement with the experimental results during steam blanching (line blue Fig. 2a). The difference between the observed and calculated results may be due to the difficulty of placing the tip of the probe at the center of the fruit. Nonetheless, in these conditions, for the holding times tested, thermal equilibrium was not reached, and the temperature profile was not homogeneous along the cross-section of the fruit (Fig. 2b). After the rapid opening of the valve and the release of the pressure, the fruits burst, and the temperature of the homogenized mash obtained after vacuum pulping reached a temperature below 35 °C for the 30 and 40 s holding times.

Previous research has shown that during the rapid release of pressure, water vapor from the fruit was instantly directed in the path of the suction of the vacuum pump, thus losing a degree of freedom of movement. During this transient period, the velocity vectors of the particles were reduced into a two-dimensional space, which significantly improved the expansion rate and therefore the vaporization and cooling rates. As this occurs without an exchange of energy with the external environment, during this transient period, the diffusion of heat and matter did not respect the classic transfer laws that prevail when the state of matter is quasistatic [18]. Therefore, during the FVE process, the temperature profile tended to approach the high-temperature short-time (HTST) process, even though the temperature gradient inside the fruit remained high, with a relatively cold zone at the heart of the fruit (Fig. 2b).

### 3.1 Microbiological quality of fruit purée obtained by FVE treatment

The results of the microbiological analysis are presented in Table 1. Goldenberry discarded from the export chain usually has a microbial load less than 3 log CFU/g for aerobic mesophilic bacteria, molds and yeasts (Experiment 1, Table 1). The 30 s steam blanching only (SB 30 s) had a poor effect on reducing microbial load, whereas FVE carried out under the same conditions (FVE 30 s) made it possible to obtain completely decontaminated purées. For steam blanching only, there was no rapid temperature drop, as shown in Fig. 2a; therefore, for the same holding time, the decontamination efficiency was expected to be higher for SB than for FVE treatment. However, this was not observed since the experimental results repeatedly showed an enhanced effect of the coupling of steam blanching and FVE on the reduction of the microbiological load despite rapid cooling and the presence of a “cold zone” at the heart of the fruit.

**Table 1:** Viability of aerobic mesophilic bacteria, molds and yeasts, and fecal and total coliforms in *Physalis peruviana* fruit purées.

Experiment 1	Plate microbial content (CFU/g)		
	Aerobic mesophilic bacteria	Molds and yeasts	Fecal and total coliforms
**Experiment 1**			
Initial count in fruits discarded from export market	2.53 × 10^2^ (±10.41)	1.43 × 10^2^ (±6.23)	<10 (±0)
SB 30 s	3.8 × 10^1^ (±6.08)	6.0 × 10^1^ (±2.0)	<10 (±0)
SB 40 s	<10 (±0)	<10 (±0)	<10 (±0)
SB 50 s	<10 (±0)	<10 (±0)	<10 (±0)
FVE 30 s	<10 (±0)	<10 (±0)	<10 (±0)
FVE 40 s	<10 (±0)	<10 (±0)	<10 (±0)
FVE 50 s	<10 (±0)	<10 (±0)	<10 (±0)
**Experiment 2**			
Initial count on spoiled fruits	1.15 × 10^6^ (±6.36)	1.61 × 10^9^ (±5.65)	9.0 × 10^6^ (±3.00)
SB 30 s	9.7 × 10^5^ (±3.66)	8.1 × 10^8^ (±5.65)	8.6 × 10^6^ (±2.56)
FVE 30 s	<10 (±0)	<10 (±0)	<10 (±0)

Mean ± standard error, n = 3**.** SB: steam blanching; FVE: flash vacuum expansion. CFU: Colony forming units. Experiment 1: Steam blanching (30, 40, and 50 s/130 kPa) with and without the application of flash vacuum expansion (5 ± 1.2 kPa). Experiment 2: logarithmic reduction of a batch of fruit at a high level of contamination and processed by heating (30 s with and without flash explosion).

Similar results were observed when applying the FVE process to other food products (Mounir et al., 2014). It is hypothesized by some authors that the rapid release of pressure and the increased rate of self-vaporization of water vapor may cause cell membrane rupture and irreversible protein denaturation [19]; they were also able to show this damage on scanning electron microscope (SEM) images of the affected microbial cells. To estimate the maximum logarithmic reduction that can be achieved by the FVE process, goldenberry fruits were allowed to spoil for several days at room temperature to reach a very high microbial load (Experiment 2, Table 1). Application of FVE to these fruits, with steam blanching for 30 s, showed minimum logarithmic reductions of 6, 9 and 7 log CFU/g for aerobic mesophilic bacteria, molds and yeasts, and fecal and total coliforms, respectively. Conversely, a very low reduction was achieved for only steam bleaching of 30 s (SB 30 s).

### 3.2 Physicochemical characterization of purée

As seen in Table 2, steam blanching alone (SB) and the FVE process significantly increased the purée production yield by a range of 15–28% compared to the nonthermally treated mash. There were no significant differences found between SB and FVE. Heat treatments soften tissues and facilitate the subsequent separation of seeds and larger fragments by sieving. A noticeable effect of the FVE process on the yield has been observed for other fruits where part of the skin was incorporated into the final puree after bursting, as in the case of purple passion fruit or grapes [10,20]. The steam blanching and FVE processes slightly increased the pH and decreased the TSS compared to the untreated purée (UP) (Table 2). However, when comparing SB to FVE, the differences in TSS were higher for blanching holding times greater than 40 s. Most likely, the bursting of the skin and tissues around the seeds solubilized some insoluble compounds that had been trapped by cell-wall polysaccharides. As shown in Fig. 2b, the skin reached the highest temperature and was, therefore, more likely to burst into small particles.

**Table 2:** Effect of steam blanching (SB) and flash-vacuum explosion (FVE) flash processes on the physicochemical variables of sieved goldenberry purées.

Variables	Steam blanching (Holding time in second)			FVE (SB Holding time in second)			UP
	30	40	50	30	40	50	
pH	3.81 ± 0.14a	3.84 ± 0.07a	3.83 ± 0.10a	3.83 ± 0.08a	3.86 ± 0.07a	3.92 ± 0.10a	3.57 ± 0.12b
Yield (% w/w)^a^	67.73 ± 0.032^a^	65.47 ± 0.04^a^	73.03 ± 0.03^a^	70.00 ± 0.03^a^	68.25 ± 0.07^a^	70.75 ± 0.03^a^	56.78 ± 0.04^b^
L*^b^	55.82 ± 1.52^a^	55.81 ± 0.35^a^	51.78 ± 0.67^b^	52.39 ± 0.24^bc^	52.24 ± 0.44^b^	53.17 ± 0.28 ^b^	51.22 ± 0.8^c^
a*^b^	25.16 ± 1.17^bc^	25.46 ± 0.44^b^	21.70 ± 0.35^e^	23.96 ± 0.31^cd^	24.42 ± 0.24^b^	23.42 ± 1.09^d^	28.45 ± 0.78^a^
b*^b^	70.22 ± 1.55^a^	69.94 ± 1.44^a^	65.23 ± 0.96^c^	65.74 ± 0.549^c^	65.35 ± 1.31^c^	67.99 ± 1.42^b^	62.73 ± 1.08^d^
ΔE*	9.53 ± 1.52a	9.08 ± 0.95a	7.32 ± 0.32b	5.67 ± 0.52c	4.93 ± 0.51c	7.64 ± 0.98b	
BI^c^	30.53 ± 0.61bc	30.88 ± 0.15bc	28.57 ± 0.11b	30.95 ± 0.34bc	31.57 ± 0.43b	29.92 ± 1.28c	36.88 ± 0.22a
SS^a^ (gxL^−1^)	12.35 ± 0.47^b^	12.20 ± 0.59^b^	10.95 ± 0.59^c^	12.82 ± 0.476^ab^	13.10 ± 1.22^ab^	12.85 ± 0.34^ab^	14.00 ± 0.15^a^
Moisture (%)	87.42 ± 0.70^a^	87.03 ± 0.31^a^	87.42 ± 0.59^ab^	86.12 ± .33^ab^	86.81 ± 0.68^bc^	85.45 ± 0.59^c^	86.30 ± 0.14 ^abc^

Mean ± standard error, n = 3, different letters in each row indicate that there is a statistically significant difference (*p* < 0.05).

a w/w: weight/weight.

b Color coordinates in the CIELAB space: L*: luminance, i.e., lightness [0–100], a*: >0: red, <0: green, b*:>0: yellow, <0: blue.

c BI=Browning Index.

a Soluble solids. FVE: flash vacuum expansion, SB: steam blanching UP: Untreated Purée.

The color coordinates of fruit purées were affected unevenly by the SB and FVE processes according to holding time. For all treatments, ΔE* was greater than 5, indicating a visually significant difference [21] compared to unpasteurized (UP) mash. However, contrary to the classic thermal treatments, ΔE* was not due to an increase in the browning index (BI), as shown in Table 2, but to an increase in the positive chromatic component b*, which represents yellowness. These changes could be attributed to the destructuring of chromoplasts and better extraction of carotenoids from the skin to the mash.

**Fig. 3:**
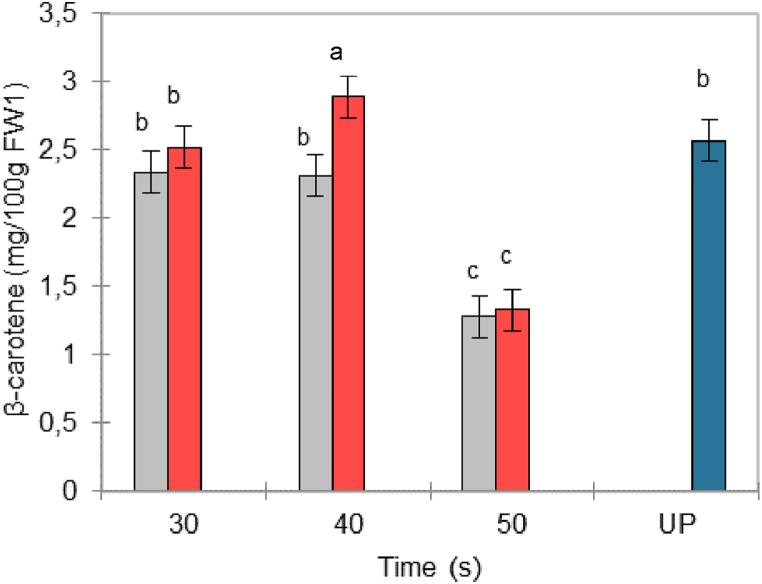
β-Carotene content in goldenberry purées obtained by steam blanching (gray bars) and flash vacuum expansion (red bars). UP: untreated purée (blue bar). ^1^FW: fresh weight. (For interpretation of the references to color in this figure legend, the reader is referred to the Web version of this article.)

Fig. 3 shows that there was a significant effect of steam blanching holding times during the FVE process on the β-carotene content. Heating times of 40 s and the application of vacuum led to a higher content of β-carotene compared to the other process treatments and inclusively UP. The 40 s treatment with FVE was able to extract carotenoids from the skin of goldenberry, which is richer in carotenoids, as shown by Etzbach et al. (2018) [16].

In addition, the values for treatments at 30 s (SB and FVE) and 40 s (SB) did not show differences with respect to the content of carotenoids found in UP (Fig. 3), indicating that under these conditions, the measurable content of β-carotene in the fruit remained stable. For a short holding time, β-carotene from goldenberry is quite heat-stable [5]; nonetheless, at 50 s, the content of carotenoids was negatively impacted due to the higher temperature reached since protein denaturation and cell wall rupture exposed carotenoids to isomerization and epoxidation reactions [22]. The results obtained for treatments at 50 s were similar to the results reported by Castro et al. (2008) for goldenberry dehydrated with hot air at 60 °C for various hours [23].

**Fig. 4:**
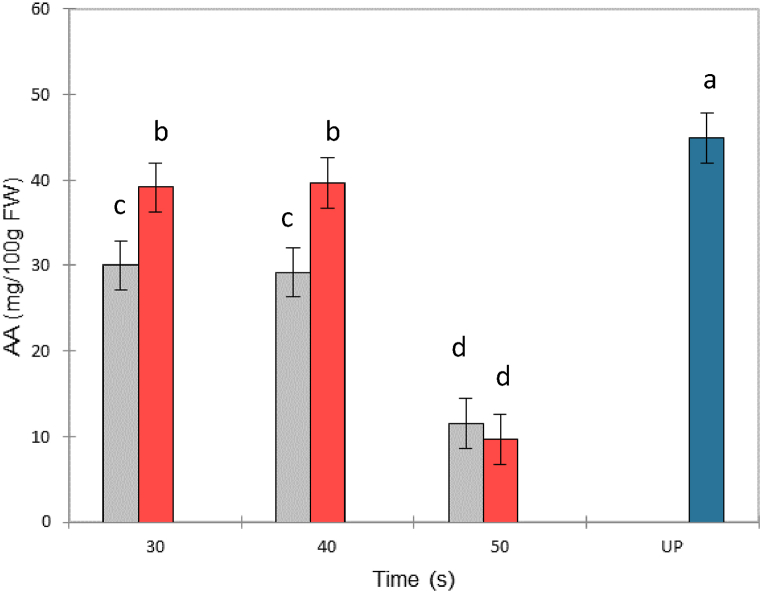
The ascorbic acid content in goldenberry purées obtained by steam blanching (gray bars) and flash vacuum expansion (red bars). UP: untreated purée (blue bar). ^1^FW: fresh weight. (For interpretation of the references to color in this figure legend, the reader is referred to the Web version of this article.)

Fig. 4 shows the effects of SB and FVE on a highly thermolabile compound, ascorbic acid (AA). The AA content (approximately 45 mg.100 g^-1^ FW) in the untreated sample (UP) was similar to that reported by Olivares-Tenorio et al. (2017) [5]. Fig. 4 shows a greater negative impact (*p* < 0.05) when only steam blanching was applied compared to the FVE process for blanching holding times of 30 and 40 s. Indeed, Fig. 2a shows that the fruit purees obtained after steam blanching and sieving at atmospheric pressure remained hot longer, whereas the addition of the FVE process induced rapid cooling, which better preserved the thermally labile ascorbic acid. Loss of AA in FVE purées with holding times of 30 and 40 s was relatively low (between 4 and 12%) with respect to blanching alone under the same conditions (approximately 30%). A similar reduction of 30% of AA has been reported for conventionally pasteurized goldenberry juice [5,24]. For 50 s blanching, a more drastic reduction (−66%) was observed, which was similar for SB and FVE, and this observation was consistent with the mash temperature history shown in Fig. 2a. The AA content was indeed a good indicator of the temperature history of the product and confirmed the impact of rapid cooling after pressure release below 40 s of steam blanching, proving again that the flash-vacuum explosion (FVE) process under these conditions better preserves thermolabile compounds. Additionally, partial vacuum during pulping prevents the oxidation and loss of bioactive compounds [25].

### 3.3 Effect of FVE on the shelf-life quality of purée

Given the analysis of the impact of FVE on the microbiological and physicochemical quality, a blanching holding time of 40 s was selected to assess the shelf-life quality of the purées produced. The goldenberry purée (FVE 40 s) was packaged following an "ultra clean" method directly at the outlet of the FVE system in irradiated multilayer aluminum bags and stored. The bioactive compounds AA and β-carotene were chosen as indicators to monitor the deterioration of FVE purées during storage at temperatures of 4 °C and 20 °C. The degradation of AA and β-carotene followed first-order kinetics, and the corresponding kinetic parameters (k, t_1/2_) are presented in Table 3. The values found for the parameter k were quite similar to pasteurized purees used for infant foods stored at 25 °C (Bosch et al., 2013). During the storage of goldenberry purée at 4 °C, there was a 12% decrease in the AA content at 30 days. After 45 days of storage, the reduction in the AA content was 80%, and after 60 days, the content remained below 10 mg·100 g^−1^. At 4 °C, the half-life of AA was much longer (t_1/2_ = 86 days) compared to storage at 20 °C (50 d). At storage of 20 °C, the reduction in AA content was much more drastic, implying that fruit purees must be kept at refrigeration temperature.

**Table 3:** Kinetic parameters of degradation of ascorbic acid and β-carotene content of cape gooseberry purée obtained by flash-vacuum explosion (40 s, 5 kPa), stored at 4 °C and 20 °C.

Compounds	Temperature (°C)	K (days−1)	R^2^	t_1/2_ (days)
Ascorbic Acid	20	0.139	0.85	50
	4	0.008	0.92	86
β-carotene	20	0.049	0.88	14
	4	0.0083	0.85	84

κ: rate constant of 1st order kinetic reaction (day^−1^); t ½: half-life time.

For β-carotene, the half-life found was 14 days for storage at 20 °C, while when using refrigeration temperatures, it was extended to 84 d. The increase in stability of β-carotene under refrigerated storage was very significant compared to the increase in stability reported for pasteurized goldenberry juice stored at 4 °C (57 d) [26]. (Reductions in β-carotene content of 38% and 51% were observed after 8 and 16 days of storage at 20 °C, respectively. After 60 days of storage at 4 °C, the residual content of carotenoids in the purée was approximately 40% of the initial content.

The results found in this study prove that the FVE process manages to produce purées with stability that is even higher than the stability reported for goldenberry extracts processed by pasteurization or HPP [27].

The high stability was also reflected in the color of stored purées. Color changes were imperceptible (ΔE*_ab_<5) up to 60 days of storage at 4 °C. The main changes were presented in the b* coordinate (yellowness) and hue angle h_ab_ (Table 4), which decreased slightly. The browning index remained constant and slightly increased between 60 and 75 days. This may be because after the process a small fraction of the PPO enzyme remains active, and its action is expressed in long storage times. According to Ali et al. (2019) phenols oxidation catalyzed by polyphenol oxidase (PPO) enzymes is known as the main factor influencing its browning [28].

**Table 4:** Color difference, chroma, and hue angle of the goldenberry purees obtained by flash-vacuum explosion (40 s, 5 kPa) during storage (4 °C).

Storage time at 4 °C	ΔE*_ab_	C_ab_	h_ab_^1^	BI^2^	b*^3^
0	–	69.04 ± 0.24a	1.37 ± 0.28 ab	32.67 ± 0.41bc	64.27 ± 0.27a
30	2.53 ± 0.65c	70.68 ± 0.84a	1.40 ± 0.03a	32.02 ± 0.03c	65.91 ± 0.88a
45	4.58 ± 0.18b	65.07 ± 0.23b	1.30 ± 0.03b	30.29 ± 0.38d	60.39 ± 0.14b
60	4.23 ± 0.71b	65.83 ± 1.29b	1.13 ± 0.02c	33.33 ± 0.43b	60.40 ± 1.08b
75	7.80 ± 0.40a	63.31 ± 0.33b	0.97 ± 0.03d	36.54 ± 0.61a	57.15 ± 0.28c

Different letters in the same column indicate significant differences(*p* < 0.05); n = 3. ^1^Hue angle. ^2^BI=Browning Index.^3^Color coordinates in the CIELAB space: b*:>0: yellow, <0: blue.

### 3.4 Analysis of energy consumption

**Fig. 5:**
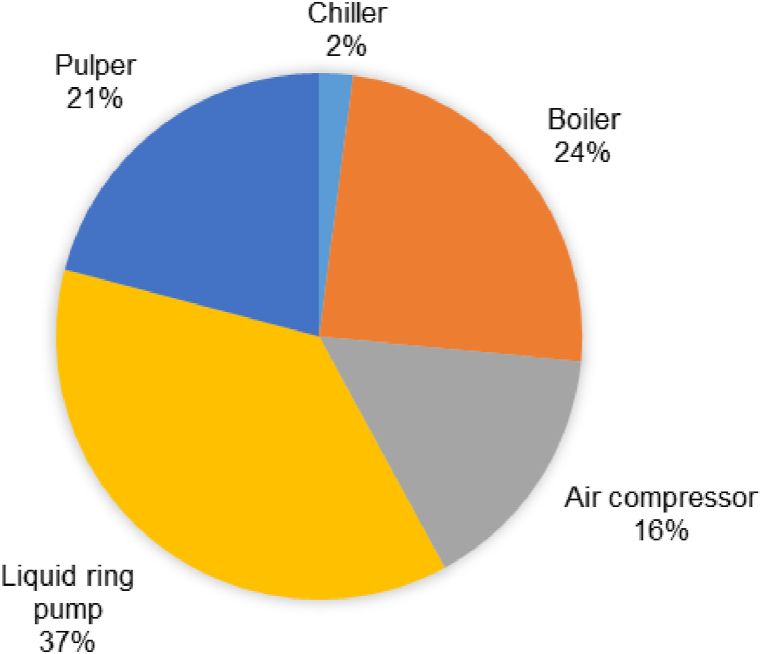
Distribution of energy consumption in the flash vacuum expansion (FVE) process.

Thus, solving equations 9, 10, 11, 12, 13, 14) yields an average specific consumption of 0.31 kWh/kg of final fruit purée. A total cycle time of 1.5 min was taken to process 1.5 kg of fresh fruit, giving an average mass throughput based on continuous processing of 64 kg of blanched whole fruit per hour, ultimately yielding 44 kg of processed mash per hour. The average specific consumption value was calculated specifically for semi-industrial pilot equipment described elsewhere [10] and could probably be reduced at a larger scale production level, as some equipment may be oversized. Nonetheless, in these conditions, the most energy-intensive operation appeared to be the liquid ring pump followed by the boiler, with power intensities of 0.11 and 0.075 kwh.kg^−1^, respectively (see Fig. 5).

Thus, the energy needs can be divided into thermal energy 0.075 kWh·kg^−1^, which can be provided by fuel oil, gas, and biogas, among others, and 0.23 kWh·kg^−1^ in electrical energy, which can also be provided by renewable sources. The specific energy consumption during the FVE process is lower on average than most conventional processes used for fruit processing, estimated at approximately 1.1 kWh.kg^−1^, including thermal and electrical energy [29]. In addition, the FVE process is carried out in one single-step 5-unit operation corresponding to blanching, pulping, pasteurization, cooling and deaeration, which requires a full range of equipment during conventional processing.

## 4 Conclusions

The process developed in this article differs from other work previously published on this topic, because in this case the FVE was directly coupled to vacuum pulping and “ultra clean” packaging giving rise to a compact line making it possible to produce a final food product without manual intervention. The study demonstrated that a very short steam blanching time (40 s) of whole fruits and the sudden application of vacuum, followed by vacuum sieving, results in a high-quality goldenberry puree. The FVE process has been shown to have an increased effect on the decontamination of goldenberry puree with a microbial reduction of more than 6 log CFU/g. Based on quality indicators such as ascorbic acid and β-carotene, the shelf life of purees was estimated at 90 days. Indeed, this innovative processing technology makes it possible to meet food safety standards without compromising the stability of the bioactive compounds. The shelf life was obtained by coupling this process with “ultra-clean” packaging, but it could probably be further extended by aseptic packaging because possible external contamination during packaging could constitute the main limitation of the shelf-life study. On the other hand, the process allows the fruit puree to be cooled almost instantaneously after the pressure drop, but although this fact is one of the main attractions of this technology, the final temperature reached is a mere observation. A better understanding of the final temperature level reached as a function of process parameters is required. Even if it is difficult to follow the temperature in real-time after the sudden drop in pressure due to the bursting of the fruits, other studies must be proposed to model and optimize the final temperature of the mash as well as the cooling slope depending on the process parameters. Finally, the equipment costs and specific energy consumption of this technology are lower than conventional processes. That kind of research is needed to evaluate the viability of the process for its adoption by SMAEs in fruit-producing areas in developing countries.

## Author contribution statement

Claudia Arias, Iris Soto: Conceived and designed the experiments; Performed the experiment; Wrote the paper.

Pablo Rodríguez, Fabrice Vaillant: Conceived and designed the experiments; Analyzed and interpreted the data; Wrote the paper.

Rowan Vaillant: Analyzed and interpreted the data; Wrote the paper

Misael Cortés: Analyzed and interpreted the data; Contributed reagents, materials, analysis tools or data; Wrote the paper.

## Patents

The authors declare that the FVE equipment used in the present work has been protect by the patent NC2021/0016741 (Resolución N° 41907, Dispositivo para la producción de purés, nectares o extractos de material vegetal. Superintendencia de industria y comercio Colombia).

## Funding

This study was financially supported by the Secretary of Agriculture (Department of Antioquia, project number 63575) in call for research 805 of the Ministry of Science, Technology, and Innovation (Minciencias-Colombia), contract number 80740-012-2019.

## Data availability statement

The data that have been used are confidential.

## Declaration of competing interest

The authors declare the following financial interests/personal relationships which may be considered as potential competing interests: Pablo Rodriguez has patent Dispositivo para la producción de purés, nectares o extractos de material vegeta issued to patent NC2021/0016741 Superintendencia de industria y comercio Colombia.

Fabrice Vaillant has patent Dispositivo para la producción de purés, nectares o extractos de material vegeta issued to patent NC2021/0016741 Superintendencia de industria y comercio Colombia.
