# Supplementary material for: Flash vacuum expansion, a low-cost and energy-efficient alternative process to produce high-quality fruit puree: Application to Physalis peruviana
Source: Heliyon. 2023 Jun 18;9(6):e16969. doi: 10.1016/j.heliyon.2023.e16969 (PMC10329112; doi:10.1016/j.heliyon.2023.e16969)
Supplement: Multimedia component 1 [file mmc1.docx]

Supplementary Content

Figure S1. Calibration curve o β-carotene by HPLC-PDA method.

R²=0.9998. Equation: Y= (4*10-5)*X + 0,7563

**Table S1.** Summary of validation parameters of β-carotene by HPLC-PDA method.

| Data | Value |
| --- | --- |
| Linear range | 1 – 30 µg/mL |
| Correlation coefficient (r^2^) | 0.9998 |
| Limit of detection (LOD) | 0.06 µg/mL |
| Limit of quantification (LOQ) | 0.18 µg/mL |
| Precision (%RSD) |  |
| Working standard solution | 57.40 µg/mL |
| Intra-day (n=6) | 57.36 µg/mL ± 0.01% |
| Inter-day (n=12) | 57.37 µg/mL ± 0.04% |
| Accuracy | 99.9% |

Figure S2. Calibration curve o ascorbic acid by HPLC-PDA method.

R²=0.9999. Equation: Y= (3.88948E-8)*X + 0.00123.

**Table S2.** Intraday and inter-day reproducibility of assay analyzed on three different days of ascorbic acid.

| Data | Value |  |  |
| --- | --- | --- | --- |
| Linear range | 1.5625 – 100 µg/mL | |  |
| Correlation coefficient (r^2^) | 0.9999 |  |  |
| Limit of detection (LOD) | 0.14 µg/mL |  |  |
| Limit of quantification (LOQ) | 0.43 µg/mL |  |  |
| Precision |  |  |  |
| Day | 1 (n=3) | 2 (n=3) | 3 (n=2) |
| Working standard solution | 15.50 µg/mL | 31.5 µg/mL | 75.0 µg/mL |
| Measurement ± RSD | 15.49 µg/mL ± 1.6% | 31.73 µg/mL ± 1.1% | 76.20 µg/mL ± 1.1% |
| Accuracy | 99.9% | 100.7% | 101.6% |
